# A local activation time histogram—An invaluable tool to diagnose a rare and complex atrial flutter mechanism

**DOI:** 10.1002/joa3.12755

**Published:** 2022-07-11

**Authors:** Abdullah Orhan Demirtas, Eduardo Sanhueza, Sheldon M. Singh

**Affiliations:** ^1^ Schulich Heart Centre, Sunnybrook Health Sciences Centre, Faculty of Medicine University of Toronto Toronto Ontario Canada

Prior ablation for atrial fibrillation (AF) can predispose patients to the development of atypical atrial flutters, which usually originate from either the left or right atria (LA or RA). Rarely, an atrial flutter circuit can actively involve both atria.^1^ Herein, we present a case of a macro‐reentrant biatrial flutter utilizing Bachmann's bundle as the critical component of the reentrant circuit.

A 70‐year‐old woman with prior history of pulmonary vein isolation, posterior wall isolation, cavotricuspid and anterior mitral flutter line placement for persistent AF presented with recurrent atrial flutter with tachycardia cycle length (TCL) of 250 ms. A local activation timing (LAT) map was created of the left atrium suggesting a breakthrough in the previously created anterior mitral line (Figure 1A, Video S1). An incomplete distribution of the tachycardia cycle length was noted in the LAT histogram. An RA LAT map was created demonstrating a region of “early‐meets‐late” in the right septum just adjacent to the earliest site in the LA (Figure 1B, Video S2). Again, an incomplete LAT histogram was noted. Careful examination of the LA and RA maps together (Figure 1C, Video S3) demonstrated that the missing portions of the LAT histogram in one chamber were present in the other suggesting the presence of biatrial flutter. We elected to target the earliest site in the LA in the region of Bachman's bundle with progressive cycle length prolongation and then termination.

**FIGURE 1:**
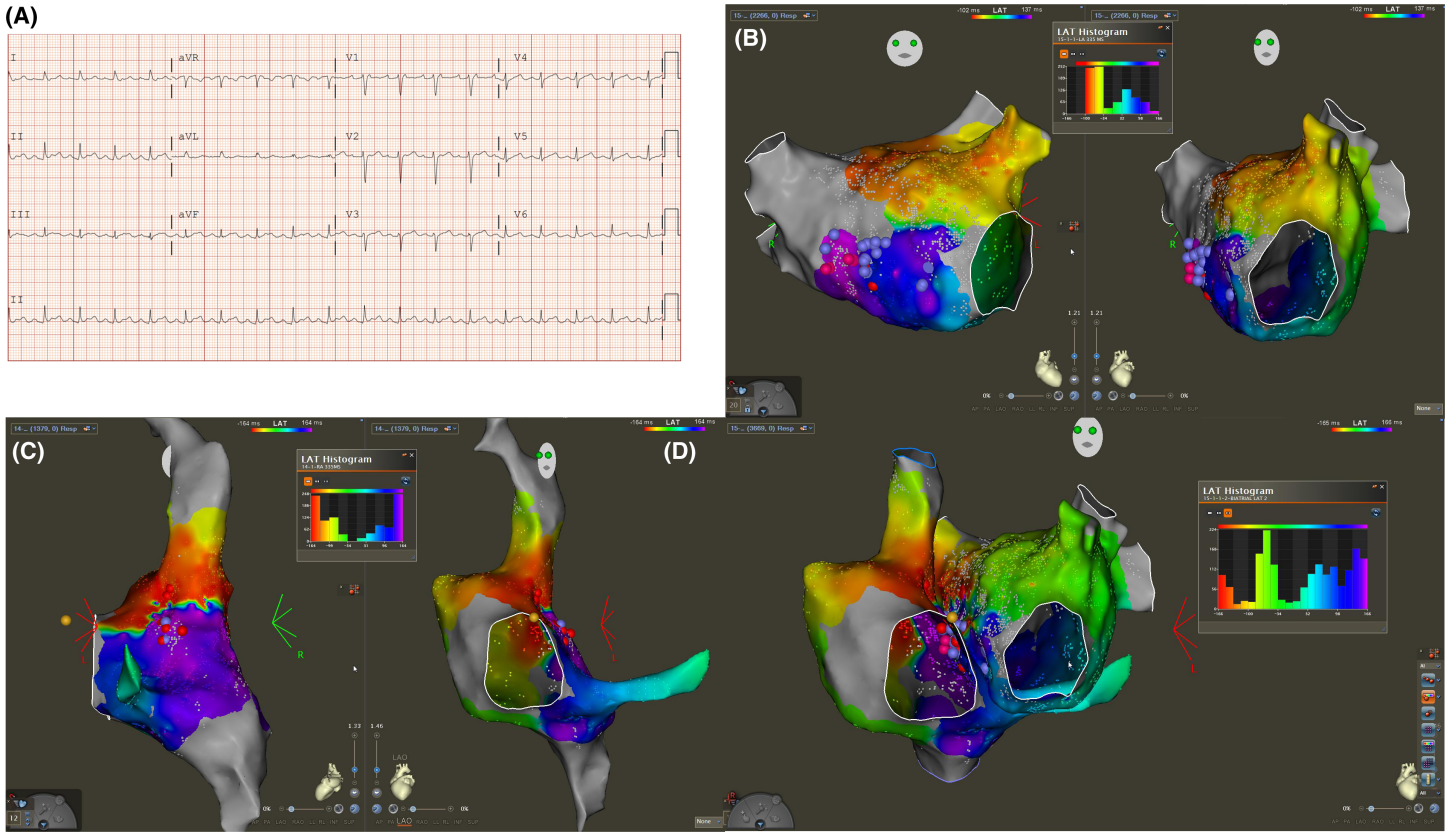
(A) Electrocardiogram in atrial flutter. (B) Left atrium local activation timing (LAT) map and histogram. (C) Right atrium LAT map and histogram. (D) Biatrial LAT map and histogram.

A biatrial flutter is a rare form of atypical flutter (0.5%–2.1%) using interatrial bridges between 2 atria.^2^ This case highlights that when all portions of an arrhythmia TCL can not be documented in one chamber, one should look for the involvement of other chambers of the heart (alternative atrium in this case, or epicardium) to better define the arrhythmia mechanism, and identify the precise region for arrhythmia termination.

## Supporting information


Video S1

Video S2

Video S3
